# The Dispensaries and Charitable Institutions of the Punjab

**Published:** 1904-05-07

**Authors:** 


### THE DISPENSARIES AND CHARITABLE INSTITUTIONS OF THE PUNJAB.

The total number of patients treated in the hospitals of the Punjab for 1902 was about 14 per cent. of the population, a slight decrease on the figures of the previous year. This was especially noticeable in the case of malarial fever and diseases of the lungs and respiratory organs, 74,000 fewer patients coming under treatment for the former, and 9,000 fewer for the latter malady. The outdoor attendance diminished very slightly, but there was a marked falling off in the number of in-patients. This is said to be owing to the prevalence of the plague, the statistics proving that any year when there is much plague in the province those who are ill avoid, as much as possible, entering a hospital. There was an increase in the number of children who were brought for treatment, but the improvement in the attendance of women which had been noted in the previous year has not been maintained in 1902. One hundred and three maternity cases with natural labour were attended to at the Lady Aitchison Hospital, Lahore, the Mayo Hospital, Lahore, and the Female Hospital, Amritsar—a strictly *purdah* institution—during the year. At the headquarters of districts there exist, in almost all cases, all the conveniences and appliances for surgery, and much good work was done throughout the province. The total of operations was 153,000, and the death-rate therefrom only .16, as against .17 in 1901.

The number of dispensaries was increased by five during 1902, and three dispensaries which were closed were replaced by new institutions in places where there was clearly a demand for them. Considerable attention has been given during the year to improvements in the Lahore Medical College. An ophthalmic ward has been added, the Mayo Hospital land has been taken up for a students' boarding house—which is much needed—and sanction has been accorded to a proposal to build separate rooms for the teaching of chemistry. The College has now reached a very satisfactory state of efficiency. With regard to income, one matter for regret is that the amount of voluntary subscriptions by natives of India for the support of dispensaries, never very large, has decreased steadily, and in 1902 only represented 1.5 per cent. of the total dispensary income. On the other hand, the proportion of the total income which is contributed by local bodies is steadily increasing, and now amounts to 91.9 per cent.

